# Prevalence of *Campylobacter* spp., *Salmonella* spp., and *Listeria monocytogenes*, and Population Levels of Food Safety Indicator Microorganisms in Retail Raw Chicken Meat and Ready-To-Eat Fresh Leafy Greens Salads Sold in Greece

**DOI:** 10.3390/foods12244502

**Published:** 2023-12-16

**Authors:** Dimitra Kostoglou, Maria Simoni, Georgios Vafeiadis, Nikolaos-Marios Kaftantzis, Efstathios Giaouris

**Affiliations:** Laboratory of Food Microbiology and Hygiene, Department of Food Science and Nutrition, School of the Environment, University of the Aegean, 81400 Myrina, Lemnos, Greece; fnsd21001@fns.aegean.gr (D.K.);

**Keywords:** foodborne bacterial pathogens, food safety indicators, raw chicken meat, fresh leafy greens salads, public health

## Abstract

The presence of microbial pathogens in foods compromises their safety resulting in foodborne illnesses, public health disorders, product recalls, and economic losses. In this work, 60 samples of chilled raw chicken meat and 40 samples of packaged ready-to-eat (RTE) fresh leafy greens salads, sold in Greek retail stores (butchers and supermarkets), were analyzed for the presence of three important foodborne pathogenic bacteria, i.e., *Campylobacter* spp., *Salmonella* spp., and *Listeria monocytogenes*, following the detection protocols of the International Organization for Standardization (ISO). In parallel, the total aerobic plate count (APC), *Enterobacteriaceae*, total coliforms, *Escherichia coli*, and staphylococci were also enumerated as hygiene (safety) indicator organisms. When present, representative typical colonies for each pathogen were biochemically verified, following the ISO guidelines. At the same time, all the *Campylobacter* isolates from chicken (*n* = 120) were identified to the species level and further phylogenetically discriminated through multiplex and repetitive sequence-based (rep) polymerase chain reaction (PCR) methods, respectively. Concerning raw chicken, *Campylobacter* spp. were recovered from 54 samples (90.0%) and *Salmonella* spp. were recovered from 9 samples (15.0%), while *L. monocytogenes* was present in 35 samples (58.3%). No *Campylobacter* was recovered from salads, and *Salmonella* was present in only one sample (2.5%), while three salads were found to be contaminated with *L. monocytogenes* (7.5%). The 65% of the *Campylobacter* chicken isolates belonged to *C. jejuni*, whereas the rest, 35%, belonged to *C. coli*. Alarmingly, APC was equal to or above 10^6^ CFU/g in 53.3% and 95.0% of chicken and salad samples, respectively, while the populations of some of the other safety indicators were in some cases also high. In sum, this study unravels high occurrence percentages for some pathogenic and food safety indicator microorganisms in raw chicken meat and RTE fresh leafy greens salads sold in Greek retail, highlighting the need for more extensive microbiological control throughout the food production chain (from the farm/field to the market).

## 1. Introduction

*Campylobacter* spp., *Salmonella* spp., and *L. monocytogenes* are important zoonotic pathogenic bacteria causing foodborne infections that can even be fatal for susceptible individuals, such as the young (fetuses, infants, and toddlers), elderly, and immunocompromised [1]. Based on the latest epidemiological data for Europe, in 2021, campylobacteriosis was the most reported foodborne gastrointestinal infection in humans in the European Union (EU) and this has been so since 2007 [2]. In 2021, campylobacteriosis corresponded to an EU notification rate of 41.1 cases per 100,000 people. Concerning salmonellosis, this remained the second most common foodborne zoonosis in humans after campylobacteriosis. That year, *Salmonella* caused 773 foodborne outbreaks (FBOs), which is the largest number of FBOs recorded for 2021. On the other hand, listeriosis concerned 2183 confirmed human cases, caused 196 deaths, and resulted in a case fatality ratio of 13.7%, which is the highest compared to that of all the other zoonoses monitored [2].

Contaminated poultry meat, especially chicken (broiler), is considered an important reservoir for both *Campylobacter* spp. and *Salmonella* spp. [3,4,5]. The high prevalence of these bacteria in such products is a result of several contamination and cross-contamination events through their production chain [6]. Nevertheless, this prevalence in poultry, as well as the contamination level of poultry products, varies greatly between different countries and product types, as well as depending on the season [7,8]. Concerning *Campylobacter* bacteria, poultry provides optimal growth conditions for them, as the physiological temperature of the birds (42 °C) coincides with the optimum temperature for the proliferation of these microaerophilic pathogens. Alarmingly, most *Campylobacter* spp. colonize and proliferate in the chicken gastrointestinal tract without any clinical symptoms, something that facilitates their transmission to humans. Among more than 30 species within the *Campylobacter* genus [9], *C. jejuni* and *C. coli* cause approximately 90% of human campylobacteriosis cases [10].

Regarding *Salmonella enterica*, this is a common pathogen that can infect both animals and humans, being a worldwide public health threat [11]. Several *Salmonella* serovars are host-specific, whereas other present a wide host range and typically cause gastroenteritis in humans. Two of the most common broad-host-range serovars associated with human illness are *S*. Enteritidis and *S*. Typhimurium; however, many other non-typhoid *Salmonella* serovars are globally isolated with great variations in prevalence still [12].

On the other hand, *L. monocytogenes* infection (i.e., listeriosis) is usually associated with the consumption of ready-to-eat (RTE) foods such as fresh produce and salads [13,14]. The source of contamination has typically been attributed to the food processing environment where *L. monocytogenes* can persist, attached to various (food contact and non-contact) surfaces, sometimes for years [15,16]. Recent outbreaks through fruits or vegetables as vehicles have raised global interest in the characterization of the public health risk due to the microbial contamination of these commodities [17,18,19]. In terms of retail, vegetables can be sold intact or minimally processed to provide an RTE product and can be contaminated at any point in the chain, starting from the farm to the plate [20]. Thus, these products can be contaminated with microbial pathogens whilst growing in fields, or during harvesting, postharvest handling, processing, and distribution. Although the prevalence of enteric pathogens, such as *Campylobacter* spp., *Salmonella* spp., and *L. monocytogenes* in RTE vegetable salads is usually low, the fact that large raw quantities of them are consumed in the last years, mainly because of an increasing demand for healthy food, without any further step of pathogen elimination in the household environment (e.g., heat treatment), dramatically increases the risk to public health [21,22]. 

Fecal indicator bacteria such as *Enterobacteriaceae*, coliforms, *E. coli*, and enterococci are routinely used to monitor the microbiological quality of water and foods [23,24]. These are microorganisms, usually non-pathogenic, that share the same habitat with enteric pathogens; that is, the gastrointestinal tract of warm-blooded animals. Thus, when such indicators are present in a sample, the latter may also be contaminated by enteric pathogens, such as *Salmonella* spp. and *L. monocytogenes*. For this reason, these bacteria are also called indicators of enteric pathogens or food safety indicators [25]. The great advantage of using such indicators is that someone does not need to check a food sample for all the possible enteric pathogens that may be present (such as enteric bacteria, viruses, protozoa, and helminths), something that would otherwise be practically and economically non-feasible. The presence of just these indicators in a quantity higher than that which would be normally expected is thus enough to treat the sample in question with concern. However, given that not all microorganisms that cause foodborne illness are enteric pathogens (for instance *Staphylococcus aureus*, *Clostridium botulinum*, and toxigenic molds), the enumeration of the total APC and/or staphylococci is also recommended. This is especially important for those food samples that could be contaminated by such non-enteric harmful microorganisms and in parallel might pose a risk to the consumer, as a more general indication of good hygienic manufacturing and processing conditions.

Although several studies have addressed the prevalence of *Campylobacter* spp., *Salmonella* spp., and *L. monocytogenes* in various types of foods consumed in Europe and in several other places throughout the world [26,27], to the best of our knowledge, there are no data on the combined prevalence of those pathogens on raw chicken meat and/or fresh leafy greens salads sold in Greece. Both these commodities are very popular to Greek consumers, as in many other countries as well. This study was therefore conducted to fill this science/epidemiological gap. Besides the detection of pathogens, all the samples (*n* = 100) were also analyzed for the populations of the total APC, *Enterobacteriaceae*, total coliforms, *E. coli*, and staphylococci, as hygiene (safety) indicator organisms, following classical microbiology procedures. Representative typical colonies for each pathogen were biochemically identified, while all the *Campylobacter* isolates (*n* = 120) were, in parallel, identified to the species level and further subtyped through classical PCR methodologies. The ultimate study aim was therefore to unravel the hygienic status of raw chicken meat and RTE fresh leafy greens salads sold in Greek retail, providing important epidemiological data for risk assessment and mitigation.

## 2. Materials and Methods

### 2.1. Sample Collection and Information on Sampling

Sixty samples of chilled raw chicken meat, including both whole carcasses, minced meat, as well as other parts of the broiler (such as breasts, thighs, wings, necks, and drumsticks) and forty samples of chilled packaged RTE fresh leafy greens salads (including vegetables such as lettuce, cabbage, radizio, rocket, carrot, and escarole) were collected from retail stores situated in Myrina, the main (capital) city of Lemnos Island. This is the 8th-largest island of Greece (among more than 200 inhabited islands) and is situated in the northeastern part of the country, with a permanent population of ca. 16.500 inhabitants, which, nevertheless, increases at least fivefold during the summer season. Analytical data on each sample type and collection retail store are presented in Tables S1 and S2 (for raw chicken meat and RTE fresh leafy greens salads, respectively). More specifically, the 20 chicken meat samples were collected from three butcher shops and the other 40 samples from three supermarkets, while all the salad samples were collected from the latter three supermarkets. All three supermarkets belong to well-known chains of stores in Greece, whose available products are circulating and consumed throughout the country. In addition, the chicken samples that were purchased from butcher shops originated from well-known poultry-producing companies whose products also circulate throughout the Greek territory. It should be noted that the three butcher shops and the three supermarkets that were visited for sample collection are all the main ones currently operating in Myrina. Regarding the chicken samples, 30% of them (*n* = 18) were prepackaged by the manufacturing industry and were all collected from the supermarkets. All chicken samples from the butchers were available for sale in bulk form. It should be stated that we could not determine in advance the exact number and type of samples to be collected from each retail store, since this was strongly dependent on the availability of the samples on the day of the visit (for collection), also considering that all the analyzed samples had to been transported to the island from continental Greece (via sea transport with refrigerated trucks). The sampling of chicken meat was performed in the period from July to October 2021, while that of salads was performed in the period from February to May 2022. All samples were provided as purchased and were immediately and individually placed in sterile sampling bags (RollBag^®^; Interscience, Saint Nom la Bretêche, France). The samples were then transported to the laboratory within one hour of purchase in cool boxes and their analysis was always executed on the same day (testing five samples each time).

### 2.2. Microbiological Analyses

#### 2.2.1. Detection and Identification of Pathogens

The samples were analyzed for the presence of *Campylobacter* spp., *Salmonella* spp., and *L. monocytogenes* following the standard ISO detection protocols, 10272-1:2017, 6579-1:2017, and 11290-1:2017, respectively [28,29,30]. For each sample, five suspect colonies (or all colonies in case there were less than five) were recovered and identified by following the biochemical tests that are reported in each ISO protocol. Thus, for *Campylobacter*, a determination of the inability of the recovered isolates to grow on *Campylobacter* Blood Agar (Biolife Italiana, Milano, Italy) at 25 °C, under aerobic conditions for 44 h, and a positive oxidase test were the two identification tests performed. For *Salmonella*, the Triple Sugar Iron Agar test was performed, together with the tests for urea hydrolysis (negative), and lysine decarboxylation (positive). For *L. monocytogenes*, tests for β-hemolysis (positive), rhamnose hydrolysis (positive), and xylose hydrolysis (negative) were executed. 

#### 2.2.2. Enumeration of Hygiene (Safety) Indicator Organisms

For the enumeration of hygiene (safety) indicator organisms, 25 g of each sample were initially homogenized with 225 mL of Maximum Recovery Diluent (MRD; Lab M, Heywood, Lancashire, UK) using a stomacher device (BagMixer^®^ 400; Interscience) for two minutes. Serial decimal dilutions were then performed by transferring 1 mL of the 1:10 homogenate (in the stomacher bag) to 9 mL of MRD in a glass tube and repeating this procedure with other tubes until reaching a 10^−^^4^ dilution. For the enumeration of APC, the pour plating method was performed with Tryptic Glucose Yeast Agar (PCA; Biolife Italiana) as the growth medium. An amount of 1 mL was used as inoculum in this method and Petri dishes were incubated at 30 °C for 72 h. For the enumeration of *Enterobacteriaceae*, the pour plating method was also performed with Violet Red Bile Glucose Agar (VRBGA; Lab M) as the growth medium, while the Petri dishes were incubated at 37 °C for 24 h. Representative typical *Enterobacteriaceae* colonies (pink to red or purple, with or without precipitation haloes) were biochemically verified through their ability to ferment glucose and their negative oxidase reaction following the ISO 21528-2 guidelines [31]. For the enumeration of total coliforms and *E. coli*, the chromogenic Harlequin™ *E. coli*/Coliform Medium (Lab M) was used and surface-inoculated with 500 μL of each suspension (10^−^^1^ till 10^−^^4^). The Petri dishes with the inoculated medium were incubated at 37 °C for 24 h. This is a selective and differential medium with *E. coli* colonies appearing as green-blue, whereas all other coliforms form pink colonies on its surface. Representative typical *E. coli* and coliform colonies from each plate were also used to inoculate Brilliant Green Bile 2% Broth (BGBB; Biolab, Budapest Hungary) with an inverted Durham tube to confirm gas production as a by-product of lactose catabolism following the ISO 4832 guidelines [32]. For the enumeration of staphylococci, the spread plating method was performed with Baird Parker Agar (Lab M) and egg yolk tellurite supplement (Lab M) as the growth medium. An amount of 0.1 mL was used as the inoculum in this method and the Petri dishes were incubated at 37 °C for 48 h [33]. Staphylococci present grey or black typical colonies on this medium (surrounded or not surrounded by opaque and/or clear zones). Such representative typical colonies (at least one from each positive sample) were also confirmed via PCR using the *Staphylococcus*-specific primers TstaG422 and Tstag765 targeting the *tuf* gene, as previously described [34]. 

### 2.3. Campylobacter Species Identification and Subtyping

#### 2.3.1. Preparation of Bacterial Cultures and Genomic DNA (gDNA) Isolation

Following their isolation from raw chicken meat samples and their purification, all the *Campylobacter* isolates (*n* = 120) were preserved long-term at −80 °C in Mueller–Hinton (MH) broth (Oxoid Limited, Thermo Fisher Scientific Inc., Waltham, MA, USA) supplemented with 5% *v*/*v* laked horse blood (HB) (Thermo Fisher Scientific Inc.) and 20% *v*/*v* glycerol (Merck KGaA, Darmstadt, Germany). Το prepare the bacterial cultures for gDNA isolation, each isolate was initially streaked on the surface of MH agar (Oxoid Limited, Thermo Fisher Scientific Inc.) and incubated under microaerophilic conditions (6.2–13.2% O_2_, 2.5–9.5% CO_2_; Oxoid™ CampyGen™ 2.5 L Sachet; Thermo Fisher Scientific Inc.) at 42 °C for 24 h (primary precultures). Following the growth of the colonies, a biomass of 5 to 10 distinct colonies was collected from the surface of the agar medium, using an inoculation loop, and then inoculated into 2 mL of fresh MH broth and incubated under microaerophilic conditions at 42 °C for 24 h (secondary precultures). Working cultures were prepared by transferring 200 μL of each secondary preculture to 1800 μL of fresh MH broth (1:10 dilution) and then incubating them under microaerophilic conditions at 42 °C for 24 h (thereby achieving a final concentration of ca. 10^8^ CFU/mL). This last procedure was repeated thrice for each isolate, and following incubation, the three Eppendorf^®^ tubes were centrifuged at 5000× *g* for 10 min at 4 °C, using a Frontier 5000 Series Multi Pro centrifuge (FC5718R, OHAUS Europe GmbH, Nänikon, Switzerland). The obtained pellets were then merged together and washed with quarter-strength Ringer’s solution (Lab M) through an additional centrifugation step.

The gDNA from each washed bacterial pellet was isolated using PureLink™ Genomic DNA Mini Kit (Thermo Fisher Scientific Inc.). The concentration of each extracted gDNA sample was determined using Qubit™ 4 Fluorometer (Thermo Fisher Scientific Inc.), while a 5 μL aliquot of it was also subjected to electrophoresis (using 1.5% *w*/*v* TBE agarose gel stained with ethidium bromide (EtBr); 100 V for 30 min) to verify its integrity, using the Mupid-One electrophoresis system (NIPPON Genetics EUROPE GmbH, Dueren, Germany) and FastGene^®^ 100 bp DNA Ladder (NIPPON Genetics EUROPE GmbH) as the molecular weight marker. At the end of electrophoresis, stained gels were visualized under UV trans-illumination using the Quantum ST4 gel documentation imaging system (Vilber Lourmat, Marne-la-Vallée, France). The rest of each gDNA sample was stored at −20 °C until its use as a substrate for the subsequent PCR reactions.

#### 2.3.2. Multiplex PCR (m-PCR) 

A previously described m-PCR (triplex) protocol for the verification of the *Campylobacter* genus and the simultaneous identification of *C. jejuni* and *C. coli* species was, here, followed [35], with some slight modifications. Briefly, Kapa Taq PCR Kit with dNTPs (F. Hoffmann-La Roche AG, Basel, Switzerland) was used for the PCRs. Each reaction mixture contained 2.5 μL of 10× kapa Taq Buffer A (1.5 mM final MgCl_2_ concentration at 1×); 0.5 μL of 10 mM dNTP Mix (0.2 mM final concentration); 0.5 μL of 10 μΜ MD16S1 primer (0.2 μM final concentration); 0.5 μL of 10 μΜ MD16S2 primer (0.2 μM final concentration); 1 μL of 10 μΜ MDmapA1 primer (0.4 μM final concentration); 1 μL of 10 μΜ MDmapA2 primer (0.4 μM final concentration); 1 μL of 10 μΜ COL3 primer (0.4 μM final concentration); 1 μL of 10 μΜ MDCOL2 primer (0.4 μM final concentration); 4 μL (200 ng) of DNA template (50 ng/μL); 0.2 μL (1 U) of Kapa Taq DNA polymerase (5 U/μL); and 12.8 μL of PCR-grade water to a total volume of 25 μL. Following their preparation, the mixtures were placed in FastGene^®^ 96-well Ultracycler (FG-TC01 Gradient version; NIPPON Genetics EUROPE GmbH). The PCR program consisted of an initial denaturation step at 95 °C for 10 min, followed by 35 cycles of denaturation at 95 °C for 30 s, primer annealing at 50 °C for 90 s, and primer extension at 72 °C for 1 min, and this was concluded by conducting a final extension step at 72 °C for 10 min. Ten μL of each PCR product were finally subjected to electrophoresis (using 1.5% *w*/*v* TBE agarose gel stained with EtBr; 100 V for 50 min) and subsequently visualized via UV trans-illumination. Genomic DNA extracted from the *C. jejuni* ATCC 33291 strain and the *C. coli* CAMP_097 chicken isolate (obtained in the present study) was always used as a positive control in each m-PCR, while the negative control that was steadily employed included PCR-grade water in the place of the substrate.

#### 2.3.3. Rep-PCR and Phylogenetic Discrimination of the Isolates

Each *Campylobacter* isolate (*n* = 107; since eight *C. jejuni* and five *C. coli* isolates failed to be resuscitated following their cryostorage for us to be able to re-extract their gDNA for the rep-PCRs) was subtyped below the species level following a previously described rep-PCR approach [36], with some minor adaptations. Briefly, Kapa Taq PCR Kit with dNTPs was used for the PCRs. Each reaction mixture contained 2.5 μL of 10× kapa Taq Buffer A; 0.5 μL of 10 mM dNTP Mix; 2.5 μL of 10 μΜ GTG_5_ primer (GTG GTG GTG GTG GTG; 1 μM final concentration); 4 μL (100 ng) of DNA template (25 ng/μL); 0.25 μL (1.25 U) of Kapa Taq DNA polymerase (5 U/μL); and 15.25 μL of PCR-grade water to a total volume of 25 μL. Following their preparation, the mixtures were placed in FastGene^®^ 96-well Ultracycler. The PCR program consisted of an initial denaturation step at 95 °C for 5 min, followed by 30 cycles of denaturation at 95 °C for 30 s, primer annealing at 40 °C for 1 min, and primer extension at 72 °C for 8 min, and this was concluded by conducting a final extension step at 72 °C for 16 min. Ten μL of each PCR product were subjected to electrophoresis (using 1.5% *w*/*v* TBE agarose gel stained with EtBr; 50 V for 2 h) and subsequently visualized via UV trans-illumination. The UV photo TIFF files of all the gels were finally treated with the open-source GelJ Java software [37]. This allowed the comparison of the rep-PCR genotypic patterns between all the isolates and the generation of phylogenetic dendrograms using the Dice coefficient and unweighted pair group method with arithmetic mean (UPGMA) cluster analysis. For this analysis, five different tolerance levels (4%, 20%, 40%, 70%, and 90%) were initially manually compared and this was finally set up to 70%, since this adjustment provided adequate discrimination power, also considering the inherent limitations of rep-PCR analysis (e.g., differences in band intensity for some of the isolates upon the replication of DNA amplification) [38]. The banding pattern of FastGene^®^ 100 bp DNA Ladder and that of *C. coli* chicken isolate CAMP_005 (obtained in the present study) were used in parallel in each rep-PCR for the normalization of the genotypic profiles for each isolate between the different experiments. 

### 2.4. Statistics

Mean values and 95% confidence intervals (C.I.) for all the prevalence data (occurrence percentages) for each surveyed pathogen were calculated and presented. The nonparametric Mann–Whitney–Wilcoxon (MWW) tests were used to test whether or not there were any significant differences in pathogen-positive raw chicken meat samples between (i) butcher shops and supermarkets, and (ii) bulk and prepackaged samples, for each pathogen separately. These were executed using the STAGRAPHICS Centurion XVI (version 16.1.11) software package (StatPoint Technologies Inc., Warrenton, VA, USA). Statistically significant differences were desired at a *p* level of <0.05.

## 3. Results

### 3.1. Prevalence of Pathogenic Bacteria in Raw Chicken Meat and RTE Fresh Leafy Greens Salads

**Figure 1:**
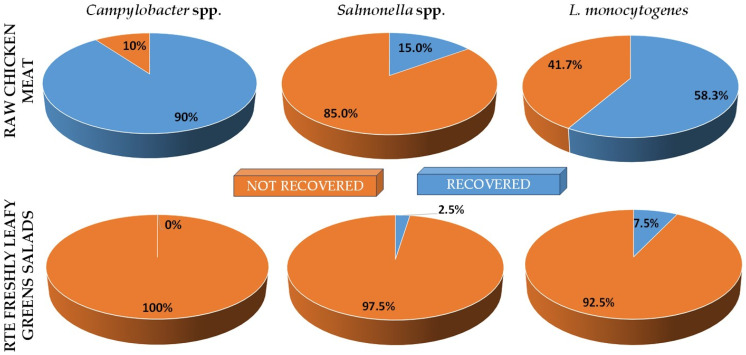
Overall prevalences (%) of *Campylobacter* spp., *Salmonella* spp., and *L. monocytogenes* in retail raw chicken meat and RTE fresh leafy greens salads collected from retail outlets in Myrina (Lemnos Island, Greece). The blue portions indicate the percentages of the pathogen-positive samples, whereas the dark orange portions indicate the percentages of the pathogen-negative samples.

The overall prevalences (%) of *Campylobacter* spp., *Salmonella* spp., and *L. monocytogenes* in raw chicken meat samples (*n* = 60) and RTE fresh leafy greens salad samples (*n* = 40) that were analyzed are depicted in Figure 1. Concerning raw chicken, *Campylobacter* spp. were recovered from 54 samples (mean prevalence: 90.0%; C.I. 95.0%: 0.82–0.98), and *Salmonella* spp. were recovered from 9 samples (mean prevalence: 15.0%; C.I. 95.0%: 0.06–0.24), while *L. monocytogenes* was present in 35 samples (mean prevalence: 58.3%; C.I. 95.0%: 0.45–0.71). No *Campylobacter* was recovered from salads, and *Salmonella* was present in only one sample (mean prevalence: 2.5%; C.I. 95.0%: −0.026–0.076), while three salads were found to be contaminated with *L. monocytogenes* (mean prevalence: 7.5%; C.I. 95.0%: −0.010–0.160). Detailed data on the presence of each pathogen in each individual chicken and salad sample are provided in Table S1 and Table S2, respectively.

Table 1 presents the prevalences (%) of pathogen-positive raw chicken meat samples, for each one of the three pathogens examined (*Campylobacter* spp., *Salmonella* spp., and *L. monocytogenes*), depending on the type of retail store (butcher shops, supermarkets) from which they were collected. Thus, 85% of samples collected from the butcher shops tested positive for *Campylobacter* spp., while that percentage was 92.5% for the samples collected from supermarkets. A higher percentage of *Salmonella* spp.-positive samples collected from supermarkets compared to those collected from butcher shops was also observed (20% and 5% positive samples, respectively). On the contrary, in the case of *L. monocytogenes*, the percentage of positive samples was lower in supermarkets compared to that of samples obtained from butcher shops (55% and 65% positive samples, respectively).

Table 2 presents the prevalences (%) of pathogen-positive raw chicken meat samples, for each one of the three pathogens examined (*Campylobacter* spp., *Salmonella* spp., and *L. monocytogenes*), depending on the sample disposal method (bulk or prepackaged). Thus, 88.1% of bulk samples tested positive for *Campylobacter* spp., while the percentage was 94.4% for the prepackaged samples. A higher percentage of *Salmonella* spp.-positive prepackaged samples than that of those disposed in bulk form was also observed (27.8% and 9.5% positive samples, respectively). On the contrary, in the case of *L. monocytogenes*, the percentage of positive samples was slightly lower when these were prepackaged compared to that of those disposed in bulk form (55.6% and 59.5% positive samples, respectively).

The statistical analyses of these prevalence results, however, revealed that neither the type of retail store nor the sample disposal method significantly influenced the recovery of pathogen-positive samples (*p* > 0.05; MWW tests).

### 3.2. Presence, Population Levels, and Frequencies of Hygiene (Safety) Indicator Organisms in Raw Chicken Meat and RTE Fresh Leafy Greens Salads

Table 3 presents aggregated data on the presence, population levels (CFU/g), and frequencies (%) of the hygiene (safety) indicator organisms in raw chicken meat samples (*n* = 60) and RTE fresh leafy greens salad samples (*n* = 40). Alarmingly, the APC was equal to or above 10^6^ CFU/g in 53.3% and 95.0% of chicken and salad samples, respectively, while at the same time, the populations of some of the other safety indicators were in some cases also high. For instance, total coliforms and *E. coli* were equal to or above 10^2^ CFU/g in 90.0% and 41.7%, respectively, of chicken samples, while staphylococci were equal to or surpassed 10^2^ CFU/g in 35.0% of salad samples. Detailed data on the population level (CFU/g) of each hygiene (safety) indicator organism in each individual chicken and salad sample are provided in Table S1 and Table S2, respectively.

### 3.3. Molecular Identification of Campylobacter Isolates and Rep-PCR Phylogenetic Analyses

**Figure 2:**
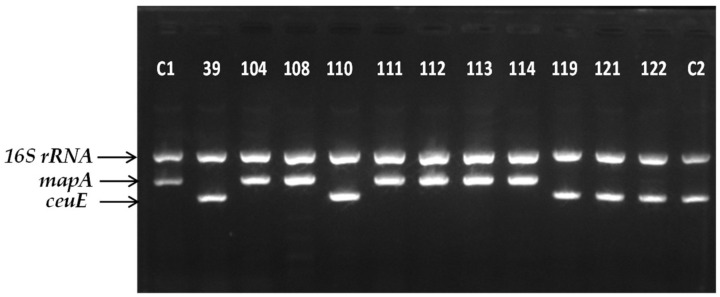
Representative banding patterns of m-PCR targeting *16S rRNA* (857 bp) gene of *Campylobacter* spp., *mapA* (589 bp) gene of *C. jejuni* (104, 108, 111, 112, 113, and 114), and *ceuE* (462 bp) gene of *C. coli* (39, 110, 119, 121, and 122) in raw chicken meat isolates. C1: *C. jejuni* ATCC 33291; C2: *C. coli* CAMP_097.

The m-PCR identification approach revealed that 65% (*n* = 78) of the *Campylobacter* chicken isolates (*n* = 120) belonged to *C. jejuni* species, whereas the other 35% (*n* = 42) concerned *C. coli* species. Interestingly, no other *Campylobacter* species were recovered. Figure 2 shows characteristic agarose electrophoresis gel presenting the m-PCR patterns of 13 *Campylobacter* spp. isolates. Thus, it is clear that the *16S rRNA* gene was successfully amplified in all the isolates (as expected), whereas the *mapA* and *ceuE* genes were solely amplified in *C. jejuni* and *C. coli* isolates, respectively.

Following the identification of the *Campylobacter* isolates to the species level (*C. jejuni*, *C. coli*), their gDNA was subjected to rep-PCR for strain differentiation (subtyping). An analysis of the derived genotypic patterns assorted the *C. jejuni* (*n* = 70) and *C. coli* (*n* = 37) isolates into eight and three groups (clusters), respectively, with a coefficient of similarity of 90% (Figure S1 and Figure S2, respectively). Two *C. jejuni* isolates (CAMP_036 and CAMP_041) and one *C. coli* isolate (CAMP_098) presented distinct rep-PCR genotypic patterns and, thus, could not be assigned in any of those clusters. It is worth noting that in many cases, isolates that originated from different chicken samples (and purchased from different retail stores) were included in the same rep-PCR group, while isolates that were included in different rep-PCR groups sometimes originated from the same chicken sample. In addition, the overall rep-PCR analysis of all the *Campylobacter* spp. isolates (*n* = 107) resulted in their classification into eight groups (clusters), again without any relationship to a species or isolation source (Figure S3). One *C. jejuni* isolate (CAMP_041) and one *C. coli* isolate (CAMP_099) could not be assigned to any of those clusters.

## 4. Discussion

In this work, the prevalence of three important foodborne pathogenic bacteria, i.e., *Campylobacter* spp., *Salmonella* spp., and *L. monocytogenes*, was investigated in two popular food products sold in Greek retail: raw chicken meat and RTE fresh leafy greens salads. Alarmingly, 95% (*n* = 57) of chicken samples were found to be contaminated by at least one of the surveyed pathogens, with 58.3% (*n* = 35) being contaminated by two pathogens in parallel and a further 5% (*n* = 3) being contaminated by all three pathogens in parallel. Thus, in these poultry products, very high prevalences were observed for *Campylobacter* spp. and *L. monocytogenes* (90% and 58.3%, respectively). The microbiological situation was, however, much better in salad samples, where only one sample (2.5%) was found to be contaminated with *Salmonella* spp., and three other samples harbored *L. monocytogenes* (7.5%). No salad samples were found to contain *Campylobacter* spp. The total absence of these latter bacteria in these commodities could be a result of their lower initial contamination level (at the farm level), the washing step that was performed at the industry level, together with the rather sensitive nature of *Campylobacter* to several adverse environmental conditions (such as freezing and desiccation) [6]. We should still mention that the relatively low number of samples that were here analyzed (*n* = 100), compared to that in other similar prevalence studies in some other countries (the most representative of which are mentioned below), together with the single collection time points and the limited geographical area of the sampling, may limit the general scalability of our findings for the whole country and overall such food products throughout the years. Surely, the enlargement of the sampling pools and time points in a future relevant study could significantly reinforce our current results. 

The legislation on the microbiological criteria for foods that is in effect in Greece is the one that applies in Europe [39]. Thus, according to EC No 1441/2007, *L. monocytogenes* should be below 100 CFU/g in all RTE products (except those intended for infants and for special medical purposes where a zero-tolerance criterion applies), such as salads, placed on the market during their shelf life. *Salmonella* spp. should be absent in 25 g of minced meat and meat preparations made from poultry meat intended to be eaten cooked. The same (strict) criterion applies to *Salmonella* spp. for precut and RTE fruit and vegetables. At the same time, regulation EC No 1086/2011 indicates an obligatory absence only of *Salmonella* serovars Typhimurium and Enteritidis in 25 g of fresh poultry meat placed on the market during its shelf life [40]. Regarding *Campylobacter* spp., the maximum limit has been set only for broiler carcasses at their manufacturing stage (after chilling) and is equal to 1000 CFU/g [41]. Thus, although these latter microaerophilic pathogens are continuously the most reported cause of foodborne gastrointestinal infection in humans in many countries worldwide, no legislation exists that concerns them in any food product placed on the EU market. 

Despite that legal omission and possibly in an attempt to fill this gap at some point, several previous studies have examined the prevalence of *Campylobacter* spp. in chicken samples sold in retail in different European countries. Thus, in an older Greek study, Lytou et al. (2020) analyzed 80 marinated chicken products obtained from meat retail markets (Attika, Greece) for the presence of *Campylobacter* spp., *Salmonella* spp., and *L. monocytogenes*, and found prevalences of 50%, 11%, and 44%, respectively, whereas 3.75% of the samples tested positive for all three pathogens [42]. In the case of *Campylobacter*, from 40 isolates in total, 27 were identified as *C. coli*, and 4 were identified as *C. jejuni*, whereas the remaining 9 belonged to unidentified species. The lower prevalence of *Campylobacter* spp. observed in that older study, compared to that in the current one, is probably attributed to the antimicrobial constituents of marinades [43], in combination with the sensitive nature of these Gram-negative non-spore-forming bacteria [44]. In another study, 17.4% of 1243 chicken meat samples that were collected from Italian supermarkets tested positive for *Campylobacter* spp., with 58% of the isolates belonging to *C. jejuni* and the rest, 42%, belonging to *C. coli* [45]. On the other hand, the prevalence of this pathogen was 73.9% in 241 samples of fresh chicken meat, at a retail store in Croatia [46]. A similar high *Campylobacter* occurrence (76%) was detected in France during an analysis that concerned 361 retail broiler meat products [47]. In that study, 64.7% and 53.1% of the products were contaminated with *C. jejuni* and *C. coli*, respectively. In another study, *Campylobacter* spp. were isolated in 32.9% of 429 broiler chicken meat samples collected from Estonian retailers [48]. Alarmingly, among the *Campylobacter*-positive samples, 6.5% contained the pathogen at concentrations above 1000 CFU/g. A similar prevalence percentage (36.7%) was recorded for *Campylobacter* spp. in retail broiler in Spain [49]. In another survey in the latter country, 39.4% of 512 retail chicken samples (half of them packed and the other half unpacked) were *Campylobacter*-positive, with unpacked products (45.3%) being more contaminated than packed ones were (33.6%) [50]. The overall prevalence of *Campylobacter* spp. in 510 raw chicken retail products sold in the Republic of Ireland was 84.3% [51]. Among the 426 isolates, 67% were *C. jejuni* and 32% were *C. coli*. *Salmonella* was also present in 5.1% of those samples. 

A few other studies have examined the prevalence of *Campylobacter* spp. in retail broilers in the USA. In a recent study, the overall prevalence of this pathogen was 36.3% in 160 chicken samples that were purchased from grocery stores in Mississippi State, remarkably without any important difference (*p* = 0.263) between conventional antibiotics-fed broilers (40.0%) and broilers raised without antibiotics (31.4%) [52]. In that study, *C. jejuni* was the predominant species, accounting for 78.1% of the isolates (*n* = 105). The prevalence of *Campylobacter* spp. in 755 skinless, boneless retail broiler meat samples collected from food stores in Alabama, from 2005 through 2011, was 41%, with no statistical differences in prevalence by year (*p* > 0.05) [53]. *C. jejuni* and *C. coli* had an average prevalence of 66% and 28%, respectively. In full agreement with the *Campylobacter* spp. prevalence that was determined in our study, these bacteria were also detected in 90% of 552 chicken meat samples that were collected from butcher shops and supermarkets over a 2-year sampling period in three Australian States [54]. In Beijing, the capital of the People’s Republic of China and the second largest city of that country, 26.3% of 240 whole chicken carcasses that were collected from the retail markets were found to be contaminated by *Campylobacter* spp., with the counts ranging from 2.5 to 7050 CFU/g [55].

In our study, *Salmonella* spp. was isolated from 15.0% (9/60) of raw chicken meat samples (*n* = 50 isolates). Given that we did not proceed with the serotyping of these isolates (which is indeed not mandatory in the ISO 6579-1:2017 detection protocol), we cannot be sure whether or not these *Salmonella*-positive samples complied with the applicable European legislation regarding fresh poultry meat (EU No 1086/2011). However, two of these samples concerned minced meat, where *Salmonella* spp. should normally be totally absent in 25 g of such products in the market, regardless of the serotype (EC No 1441/2007). It should still also be noted that the ISO protocol defines the testing of colonies showing typical biochemical reactions for *Salmonella* for the presence of *Salmonella* O- and H- antigens. Unfortunately, in this research, we did not proceed with this serological verification due to time and financial constraints. This is hopefully planned to be carried out in the future. Many other studies have analyzed the prevalence of this pathogen in retail broilers sold in Europe and elsewhere. In an older such Greek study, *Salmonella* spp. were isolated from 39.5% of 96 chicken carcasses from 22 different commercial farm brands found in the retail market [56]. Interestingly, a significantly higher isolation rate (60.4%) was observed during the summer (May to October), compared to that during the winter (18.7%; November to April). In another similar study in Spain, *Salmonella* spp. were isolated from 35.8% of 198 samples of chicken meat (legs) for sale in retail outlets and supermarkets [57]. Thermophilic campylobacters were also isolated in 49.5% of those samples. A similar *Salmonella* prevalence (31.5%) was also observed during the analysis of 698 chicken carcasses (both chilled and frozen) that were collected from retail stores in three different regions of Russia [58]. In another study in China, among 200 retail raw chicken carcasses (both chilled and frozen) that were purchased in wet markets and supermarkets in Shaanxi Province, 46.5% were *Salmonella*-positive [59]. In addition, those results revealed that the pathogen was more prevalent in samples during the spring and summer than during the autumn and winter. Similarly, the overall prevalence of *Salmonella* spp. in chilled chicken meat (115 samples) sold at retail in the Federal District, Brazil, was 46.1% [60]. In another study in Latin America, this time in Mexico, *S. enterica* was recovered from 18.1% of 1160 samples that were collected from wet markets and supermarkets [61]. Remarkably, during the three years of that survey, the pathogen was more dominant in supermarkets (27.2%) than in wet markets (9.0%). 

*L. monocytogenes* is an important foodborne pathogen, characterized by high hospitality and mortality rates [62]. This is mostly associated with RTE food categories that are preserved under refrigeration. In Europe, and based on the latest epidemiological data for 2021, the highest occurrence values (from 2% to 5%) were observed for fish and fishery products, meat products (from bovines or pigs), and cheeses (from sheep milk), while the overall occurrence in fruits and vegetables (*n* = 1407) was also high at 3.0% [2]. However, for all food categories, the proportion of samples exceeding the limit of the legislative criteria upon distribution (100 CFU/g) was (hopefully) low (0% to 0.66%). In our study, this pathogen was detected in 58.3% of raw chicken samples (35/60) and in 7.5% of RTE fresh leafy greens salad samples (3/40). Worldwide, there are a few studies published examining the prevalence of *L. monocytogenes* in raw chicken meat. In a study conducted in 2002 in the two largest cities in Estonia (Tallinn and Tartu), 240 raw broiler legs in total (half from Estonia and the other half of foreign origin) were collected from 12 retail stores. Of these, 70% were positive for *L. monocytogenes*, with the prevalence of the pathogen being significantly higher in samples of Estonian origin (88%) than that in those of foreign origin (53%) [63]. In a more recent study conducted in 2020 in Egypt, the prevalence of *L. monocytogenes* in 75 fresh retail chicken meat samples was 48% [64]. Another study evaluated the prevalence of *L. monocytogenes* in 552 refrigerated samples of ground beef, chicken leg (*n* = 138), hot dog, and pork sausage collected in supermarkets in the city of Sao Paulo, Brazil [65]. The pathogen was detected in 48.7% of the samples, with the highest prevalence being in ground beef (59.4%) followed by chicken legs (58.0%), pork sausages (39.8%), and hot dogs (37.7%). Perhaps relievedly, the populations were below 100 CFU/g in most of those samples (62.5%). On the other hand, Goh et al. (2012) determined a 20% prevalence for *L. monocytogenes* in raw chicken meat samples (*n* = 210) at wet markets and hypermarkets in Malaysia [66]. Interestingly, the pathogen occurred more frequently in samples from hypermarkets (25.7%) compared to those from wet markets (14.3%).

The minimal processing of leafy greens typically concerns the elimination of external wilted or ruined leaves, cutting, washing, drying, and packaging. Those procedures not only result in an RTE, convenient product but also ensure the preservation of the vegetables’ organoleptic properties. However, this form of processing generally results in a shorter shelf life compared to that of the starting product. Thus, the average shelf life of RTE salads generally ranges from 5 to 7 days (at temperatures below 8 °C), and this is reduced to a maximum of 2 days after the opening of the packages [67]. To extend shelf life, in recent years, modified atmosphere packaging (MAP) has been employed by many fresh produce industries, and several others as well [68]. However, the main issue still associated with these products is the potentially high microbiological risk since these are consumed as purchased with no application of any further pathogen elimination step in the household environment. Surely, the contamination of vegetables growing in soil with a plethora of microorganisms is something common and inevitable. This, in combination with their high water activity value (a_w_ > 0.98), permissive pH (6.0–7.0), possibility of unintended temperature abuse (during processing, transportation, and storage), and lack of stringent decontamination procedures, increase the risk for pathogen transmission associated with these products [21,22]. Not surprisingly therefore, several studies have been published on the prevalence of foodborne pathogens in RTE salads.

In a predictive risk assessment study in the Netherlands, over 1800 samples of raw produce (13 types of vegetables) and over 1900 samples of the resulting RTE mixed salads were investigated for *S. enterica* serovars, *Campylobacter* spp., *E. coli* O157, and *L. monocytogenes* [69]. Only one retail sample was found to be positive for *Salmonella* Montevideo. The overall prevalence point estimates for the microorganisms in raw produce varied from 0.11% for *L. monocytogenes* and *E. coli* O157, and 0.22% for *Campylobacter* spp. to 0.38% for *Salmonella*. In agreement with our results, in another surveillance study involving a range of retail food samples (*n* = 2391) that were purchased in three Irish cities, *Campylobacter* spp. were not detected in any of the vegetables and prepared salads (*n* = 62) that were examined [70]. Arienzo et al. (2020) evaluated the microbiological quality and safety of two different varieties of RTE salads (baby romaine lettuce and rocket) sold in widespread supermarket chains in Italy, on the packaging date, during the shelf life, and during home refrigeration [67]. All batches (*n* = 16) were compliant with European standards for *L. monocytogenes* (<100 CFU/g); conversely 67% of those tested positive for *Salmonella* spp., resulting in a non-compliant status regarding the European regulation (EC No 1441/2007). All the *Salmonella*-positive batches were found to be positive both on the packaging and expiry date. Remarkably, of all the samples analyzed on the packaging date, only 17% displayed a total aerobic mesophilic count that was below 10^6^ CFU/g. In another study in Croatia, *L. monocytogenes* was detected in only one sample (1%) of cut red cabbage (in a population of less than 100 CFU/g) during the analysis of 100 samples of RTE vegetables collected from supermarkets [71]. In another study in central Ohio (Columbus, OH, USA), in total, 364 samples of salad vegetables (leafy greens, tomatoes, and cucumbers) were collected from farmers’ markets and grocery stores [72]. No *Salmonella* spp. or carbapenem-resistant *Enterobacteriaceae* were recovered. The mean coliform counts differed (*p* < 0.05) between produce types, with the count in tomatoes (15 CFU/mL) being lower than that in cucumbers (77.4 CFU/mL) and leafy greens (75.0 CFU/mL). There was no difference in coliform counts in produce purchased from farmers’ markets and grocery stores.

Besides the detection of the three pathogens, all raw chicken meat and salad samples were also analyzed in the present study for the populations (CFU/g) of the total APC, *Enterobacteriaceae*, total coliforms, *E. coli*, and staphylococci, as a more general indication of their overall microbiological quality. Thus, although no mandatory microbiological criteria exist in Europe that include the evaluation of those microbiological parameters, several national guidelines still take them into account as indicators of the overall microbiological quality of foods’ production processes. However, these latter guidelines mostly concern RTE food categories. For instance, the Food Safety Authority of Ireland (FSAI) has set limits for *Enterobacteriaceae* and *E. coli* in RTE foods placed on the market of <10^2^ CFU/g and <20 CFU/g, respectively [73]. Those guidelines also set a maximum limit for coagulase-positive staphylococci of 20 CFU/g. The same limit for *Enterobacteriaceae* in RTE foods (<10^2^ CFU/g) is also proposed by the UK Health Protection Agency (now Health Security Agency, Canary Wharf, London, UK) [74]. On the other hand, the Canadian Health Authority defines the limits for coliforms and *E. coli* as <10^2^ CFU/g and <10 CFU/g, respectively [75]. Alarmingly, in our survey, the APC was equal to or above 10^6^ CFU/g in 53.3% and 95.0% of chicken and salad samples, respectively, while the populations of some of the other safety indicators in some cases could also be considered high. For instance, in 10% of the salad samples (4/40), the population of *Enterobacteriaceae* was equal or exceeded 10^4^ CFU/g.

Rep-PCR-based typing with the GTG_5_ primer is a rapid, simple, cost-efficient, and easy-to-perform method that shows high discriminatory power. As a result, several previous studies have been published that used this method for the quick discrimination of strains of various bacterial species [76,77]. In addition, like us, a few previous studies have also exploited this genotyping method for the clustering of *Campylobacter* spp. isolates. For instance, a previous study has used GTG_5_-PCR and pulsed-field gel electrophoresis (PFGE) in parallel for the typing of 72 epidemiologically independent *C. jejuni* strains [38]. Interestingly, both methods presented identical discriminatory power, with the rep-PCR assay being considerably more rapid and economical, and simpler. In another study, rep-PCR, using the primer pairs ERIC and GTG_5_ in parallel in two different reactions, was compared to the widely used multilocus sequence typing (MLST) for the differentiation of 16 *C. jejuni* isolates from broilers [78]. Both techniques demonstrated equal discriminatory power. Behringer et al. (2011) analyzed 100 *Campylobacter* spp. isolates (*C. jejuni* and *C. coli*) from live broilers and retail broiler meat with four molecular typing methods: restriction fragment length polymorphism of the *flaA* gene (*flaA*-RFLP), MLST, PFGE, and automated rep-PCR (using the DiversiLab system) [79]. All methods performed similarly for the typing of those isolates. The rep-PCR method was better for the typing of *C. jejuni* than that of *C. coli*, while the generated patterns appeared to be random, without any relationship to species, location, or source of isolates. Such a lack of correlation of derived rep-PCR clusters to the *Campylobacter* species or isolation source was also observed in the present study.

## 5. Conclusions and Perspectives

The high occurrence percentages of some pathogenic bacterial species that were observed in the present study in the raw chicken meat samples (90.0% and 58.3%, for *C. jejuni/coli* and *L. monocytogenes*, respectively) surely highlight the need for more extensive microbiological control throughout their full production chain (from the farm to the market). These, in parallel, suggest that preventive measures such as good husbandry conditions in poultry facilities, the more stringent application of the hazard analysis of critical control points (HACCP) system mainly at the industry and retail levels, and consumer education on the proper handling of raw poultry during preparation and cooking should be reinforced to ensure food safety. On the other hand, although all the three surveyed pathogens (*Campylobacter* spp., *Salmonella* spp., and *L. monocytogenes*) were found to present lower prevalences in the RTE fresh leafy greens salad samples (0.0%, 2.5%, and 7.5%, respectively), the high counts for total APC, *Enterobacteriaceae*, and staphylococci that were enumerated in several of those samples surely advocate for the further optimization of their production process again beginning from the field. Soon, the examination of the pathogenic isolates (mainly *Campylobacter* spp.) for their antibiotic resistance, virulence potential, and biofilm forming abilities, together with the whole genome sequencing (WGS) of some of them, is planned to be executed by our team for us to obtain more information on their potential risk and, in parallel, to try to unravel any relationship between some of those phenotypes and genomic profiles, possibly looking for any relevant important genetic biomarkers.

## Figures and Tables

**Table 1:** Prevalences (%) of pathogen-positive raw chicken meat samples, for each one of the three pathogens examined (*Campylobacter* spp., *Salmonella* spp., and *L. monocytogenes*), depending on the type of retail store (butcher shops and supermarkets) from which they were collected.

Type of Retail Store			*Campylobacter* spp.			*Salmonella* spp.			*L. monocytogenes*		
	Total Samples		Positive Samples			Positive Samples			Positive Samples		
	*n*	%	*n*	%		*n*	%		*n*	%	
				In Each Type of Retail Store	Of Total		In Each Type of Retail Store	Of Total		In Each Type of Retail Store	Of Total
Butcher shops	20	33.3%	17	85.0%	31.5%	1	5.0%	11.1%	13	65.0%	37.1%
Supermarkets	40	66.7%	37	92.5%	68.5%	8	20.0%	88.9%	22	55.0%	62.9%
Sum	60	100.0%	54	90.0%	100.0%	9	15.0%	100.0%	35	58.3%	100.0%

**Table 2:** Prevalences (%) of pathogen-positive raw chicken meat samples, for each one of the three pathogens examined (*Campylobacter* spp., *Salmonella* spp., and *L. monocytogenes*), depending on the sample disposal method (bulk or prepackaged).

Sample Disposal Method			*Campylobacter* spp.			*Salmonella* spp.			*L. monocytogenes*		
	Total Samples		Positive Samples			Positive Samples			Positive Samples		
	*n*	%	*n*	%		*n*	%		*n*	%	
				In Each Disposal Method	Of Total		In Each Disposal Method	Of Total		In Each Disposal Method	Of Total
Bulk	42	70.0%	37	88.1%	68.5%	4	9.5%	44.4%	25	59.5%	71.4%
Prepackaged	18	30.0%	17	94.4%	31.5%	5	27.8%	55.6%	10	55.6%	28.6%
Sum	60	100.0%	54	90.0%	100.0%	9	15.0%	100.0%	35	58.3%	100.0%

**Table 3:** Presence, population levels (CFU/g), and frequencies (%) of hygiene (safety) indicator organisms (APC, *Enterobacteriaceae*, total coliforms, *E. coli*, and staphylococci) in raw chicken meat and RTE fresh leafy greens salads.

Hygiene (Safety) Indicator Organism	Population Level (CFU/g) and Frequencies (%)	
	Raw Chicken Meat (*n* = 60)	RTE Freshly Leafy Greens Salads (*n* = 40)
APC	≥10^6^ CFU/g in 32 samples (53.3%)	≥10^6^ CFU/g in 38 samples (95.0%)
*Enterobacteriaceae*	≥10^4^ CFU/g in 19 samples (31.7%)	≥10^4^ CFU/g in 4 samples (10.0%)
Total coliforms	≥10^2^ CFU/g in 54 samples (90.0%)	≥10^2^ CFU/g in 0 sample (0.0%)
*E. coli*	≥10^2^ CFU/g in 25 samples (41.7%)	≥10^2^ CFU/g in 0 sample (0.0%)
Staphylococci	≥10^2^ CFU/g in 6 samples (10.0%)	≥10^2^ CFU/g in 14 samples (35.0%)

## Data Availability

The data presented in this study are contained within the article or Supplementary Materials.

## Supplementary Materials

The following supporting information can be downloaded at https://www.mdpi.com/article/10.3390/foods12244502/s1. Table S1: Presence of pathogens (*Campylobacter* spp., *Salmonella* spp., and *L. monocytogenes*) and population levels (CFU/g) of hygiene (safety) indicator organisms (APC, *Enterobacteriaceae*, total coliforms, *E. coli*, and staphylococci) in each raw chicken meat sample (*n* = 60); Table S2: Presence of pathogens (*Campylobacter* spp., *Salmonella* spp., and *L. monocytogenes*) and population levels (CFU/g) of hygiene (safety) indicator organisms (APC, *Enterobacteriaceae*, total coliforms, *E. coli*, and staphylococci) in each RTE fresh leafy greens salad sample (*n* = 40); Figure S1: Cluster analysis (dendrogram) based on rep-PCR genotypic patterns of *C. jejuni* raw chicken meat isolates (*n* = 70). Each separate group (B, C, D, F, G, H, I, and J) includes isolates with a coefficient similarity of above 90%. The origin of each isolate (sample s/n) is also indicated. The *C. jejuni* ATCC 33291 strain is indicated with the arrow; Figure S2: Cluster analysis (dendrogram) based on rep-PCR genotypic patterns of *C. coli* raw chicken meat isolates (*n* = 37). Each separate group (a, c, and d) includes isolates with a coefficient similarity of above 90%. The origin of each isolate (sample s/n) is also indicated; Figure S3: Cluster analysis (dendrogram) based on rep-PCR genotypic patterns of *Campylobacter* spp. in raw chicken meat isolates (*n* = 107). Each separate group (B, C, D, E, F, G, H, I, and J) includes isolates with a coefficient similarity of above 90%. The origin of each isolate (sample s/n) is also indicated.
