# Supplementary material for: Prevalence of Campylobacter spp., Salmonella spp., and Listeria monocytogenes, and Population Levels of Food Safety Indicator Microorganisms in Retail Raw Chicken Meat and Ready-To-Eat Fresh Leafy Greens Salads Sold in Greece
Source: Foods. 2023 Dec 16;12(24):4502. doi: 10.3390/foods12244502 (PMC10742679; doi:10.3390/foods12244502)
Supplement: Supplementary file 1 [file foods-12-04502-s001.zip › foods-2742178-supplementary.pdf]

Table S1. Presence of pathogens (*Campylobacter* spp., *Salmonella* spp., and *L. monocytogenes*) and population levels (CFU/g) of hygiene (safety) indicator organisms (APC, *Enterobacteriaceae*, total coliforms, *E. coli*, and staphylococci) in each raw chicken meat sample ($n = 60$).

s/n	Sample type	Sample disposal method	Retail store	Presence (detection)			Population level (CFU/g)				
				Campylobacter spp.	*Salmonella* spp.	*L. monocytogenes*	APC	*Enterobacteriaceae*	Coliforms	*E. coli*	Staphylococci
1	minced meat	bulk	supermarket #1	+	+	+	2.48 x 10^7	2.48 x 10^4	2.80 x 10^3	2.20 x 10^2	1.00 x 10^2
2	breast	prepackaged	supermarket #2	+			1.20 x 10^6	1.90 x 10^3	5.80 x 10^2	5.40 x 10^2	< 10^2
3	fillet	bulk	supermarket #3	+			3.10 x 10^6	7.90 x 10^3	2.80 x 10^3	8.00 x 10^2	1.00 x 10^2
4	thigh	bulk	butcher shop #1	+			1.71 x 10^6	4.50 x 10^3	4.00 x 10^1	4.00 x 10^1	< 10^2
5	breast (from whole carcass)	bulk	butcher shop #2	+		+	1.04 x 10^6	1.96 x 10^5	2.46 x 10^3	8.00 x 10^1	< 10^2
6	wings	bulk	butcher shop #1	+			1.64 x 10^5	1.02 x 10^5	2.48 x 10^3	1.72 x 10^3	1.00 x 10^2
7	neck - platars	prepackaged	supermarket #2	+			2.44 x 10^6	5.16 x 10^5	2.60 x 10^3	2.60 x 10^3	< 10^2
8	thigh	bulk	supermarket #3	+			2.39 x 10^5	2.20 x 10^4	1.80 x 10^2	< 10	< 10^2
9	fillet	bulk	supermarket #1	+			1.38 x 10^5	2.80 x 10^4	2.20 x 10^2	< 10	< 10^2
10	minced meat	bulk	butcher shop #1	+	+		3.20 x 10^7	7.80 x 10^4	9.80 x 10^3	< 10	< 10^2
11	thigh stripes	prepackaged	supermarket #2	+		+	6.70 x 10^5	6.90 x 10^2	6.00 x 10^2	8.00 x 10^1	< 10^2
12	breast	bulk	supermarket #3				8.70 x 10^5	2.50 x 10^3	1.06 x 10^4	6.00 x 10^1	1.00 x 10^2
13	wings	prepackaged	supermarket #1	+		+	6.50 x 10^5	2.80 x 10^3	1.02 x 10^4	2.00 x 10^2	< 10^2
14	thigh (from whole carcass)	bulk	butcher shop #2	+		+	1.08 x 10^5	3.00 x 10^1	< 20	< 10	< 10^2

15	necks (from whole carcass)	bulk	butcher shop #2	+		+	8.28 x 10^7	1.06 x 10^5	4.06 x 10^5	1.40 x 10^3	1.00 x 10^2
16	thigh fillet	bulk	supermarket #1	+		+	1.47 x 10^7	2.01 x 10^4	3.78 x 10^4	6.00 x 10^1	< 10^2
17	thigh	bulk	butcher shop #1			+	3.60 x 10^4	8.00 x 10^1	6.00 x 10^1	< 10	< 10^2
18	stomachs	prepackaged	supermarket #2	+	+		8.60 x 10^5	2.00 x 10^1	4.80 x 10^2	< 10	< 10^2
19	minced meat	bulk	butcher shop #1	+		+	2.34 x 10^8	2.02 x 10^5	1.38 x 10^6	2.00 x 10^1	< 10^2
20	wings	bulk	butcher shop #1	+		+	8.00 x 10^6	8.40 x 10^3	4.04 x 10^3	2.00 x 10^2	< 10^2
21	wings	bulk	supermarket #1	+		+	1.53 x 10^7	3.85 x 10^4	1.56 x 10^4	8.00 x 10^1	< 10^2
22	breast	prepackaged	supermarket #2	+		+	1.44 x 10^6	7.60 x 10^2	2.16 x 10^3	2.00 x 10^1	< 10^2
23	thigh	bulk	supermarket #3	+	+		9.44 x 10^7	2.69 x 10^5	2.13 x 10^6	1.56 x 10^3	< 10^2
24	breast (from whole carcass)	bulk	butcher shop #3	+			5.20 x 10^4	2.50 x 10^2	1.40 x 10^2	< 10	< 10^2
25	souvlaki	bulk	supermarket #1	+		+	8.68 x 10^7	5.90 x 10^3	5.60 x 10^3	1.60 x 10^2	< 10^2
26	viscera	prepackaged	supermarket #2	+	+		2.50 x 10^6	1.30 x 10^4	1.36 x 10^4	1.60 x 10^2	< 10^2
27	breast	bulk	butcher shop #1	+		+	1.93 x 10^6	6.40 x 10^4	1.56 x 10^3	4.00 x 10^1	< 10^2
28	thigh (from whole carcass)	prepackaged	supermarket #2	+			4.00 x 10^3	1.00 x 10^1	2.00 x 10^1	< 10	< 10^2
29	platar (from whole carcass)	prepackaged	supermarket #2	+		+	9.90 x 10^5	2.50 x 10^2	2.80 x 10^2	1.60 x 10^2	< 10^2
30	wings	prepackaged	supermarket #1	+	+		1.88 x 10^5	1.06 x 10^3	3.16 x 10^3	2.00 x 10^2	< 10^2
31	bone-in breast	bulk	supermarket #3	+			1.90 x 10^4	1.80 x 10^2	3.00 x 10^2	4.00 x 10^1	< 10^2
32	breast fillet skewer	bulk	butcher shop #1			+	8.40 x 10^5	7.90 x 10^2	5.00 x 10^2	< 10	< 10^2
33	thigh	bulk	supermarket #1	+		+	2.34 x 10^7	1.70 x 10^4	1.18 x 10^5	< 10	< 10^2

34	platars	prepackaged	supermarket #2	+		+	8.00 x 10^6	6.50 x 10^3	1.22 x 10^4	2.80 x 10^3	1.00 x 10^2
35	neck - platar (from whole carcass)	bulk	supermarket #1	+		+	2.87 x 10^5	6.50 x 10^3	2.66 x 10^3	1.40 x 10^4	< 10^2
36	breast (from whole carcass)	prepackaged	supermarket #1				1.00 x 10^3	< 10	< 20	< 10	< 10^2
37	minced meat	bulk	butcher shop #1	+			2.10 x 10^6	2.58 x 10^3	2.24 x 10^3	1.00 x 10^2	< 10^2
38	wings	bulk	supermarket #3	+		+	6.16 x 10^7	1.12 x 10^4	8.80 x 10^4	1.00 x 10^2	1.70 x 10^3
39	breast fillet	bulk	supermarket #3	+		+	4.14 x 10^7	1.43 x 10^4	3.18 x 10^4	1.88 x 10^3	< 10^2
40	thigh	bulk	butcher shop #1	+		+	9.70 x 10^6	1.89 x 10^4	2.18 x 10^4	< 10	< 10^2
41	thigh	prepackaged	supermarket #2	+		+	4.10 x 10^5	2.20 x 10^2	6.20 x 10^2	1.00 x 10^2	< 10^2
42	wings	bulk	supermarket #1	+		+	6.04 x 10^7	4.00 x 10^4	2.86 x 10^5	1.20 x 10^2	< 10^2
43	breast fillet	prepackaged	supermarket #1	+		+	3.10 x 10^5	8.90 x 10^2	6.60 x 10^2	1.40 x 10^2	< 10^2
44	souvlaki (with green pepper between the pieces)	bulk	supermarket #1	+		+	8.08 x 10^7	3.96 x 10^3	4.72 x 10^3	1.20 x 10^2	< 10^2
45	wings	prepackaged	supermarket #2	+	+	+	1.78 x 10^6	1.62 x 10^3	4.72 x 10^3	2.00 x 10^2	< 10^2
46	minced meat	bulk	butcher shop #1	+		+	1.43 x 10^6	1.59 x 10^3	1.06 x 10^3	< 10	< 10^2
47	thigh	bulk	supermarket #3	+			1.34 x 10^6	4.00 x 10^1	2.00 x 10^2	< 10	< 10^2
48	breast (from whole carcass)	bulk	butcher shop #2	+			2.53 x 10^5	1.50 x 10^2	1.40 x 10^2	4.00 x 10^1	< 10^2
49	wings	bulk	butcher shop #1			+	5.30 x 10^4	5.30 x 10^2	4.00 x 10^2	2.00 x 10^1	< 10^2
50	minced meat	bulk	supermarket #1	+		+	1.48 x 10^5	5.40 x 10^3	6.20 x 10^4	1.80 x 10^2	< 10^2
51	platars	prepackaged	supermarket #2	+	+	+	8.40 x 10^3	1.10 x 10^2	2.60 x 10^2	6.00 x 10^1	< 10^2
52	thigh (from whole carcass)	bulk	butcher shop #2	+			1.60 x 10^5	< 10	2.00 x 10^1	< 10	< 10^2

53	drumsticks	prepackaged	supermarket #2	+		+	1.41 x 10^8	4.70 x 10^3	9.84 x 10^4	3.20 x 10^2	< 10^2
54	souvlaki (with green pepper between the pieces)	bulk	supermarket #1	+	+		3.72 x 10^7	2.70 x 10^3	2.56 x 10^3	2.00 x 10^1	< 10^2
55	thigh	bulk	supermarket #3				3.10 x 10^6	6.00 x 10^2	4.00 x 10^3	4.00 x 10^1	< 10^2
56	breast fillet (cut into bites)	bulk	butcher shop #1	+		+	3.10 x 10^7	9.30 x 10^3	1.40 x 10^4	4.00 x 10^1	< 10^2
57	minced meat	bulk	butcher shop #1	+		+	9.60 x 10^5	6.30 x 10^2	6.00 x 10^2	2.00 x 10^1	< 10^2
58	thigh	prepackaged	supermarket #2	+			2.85 x 10^5	4.90 x 10^2	1.08 x 10^3	8.00 x 10^1	< 10^2
59	breast	bulk	supermarket #3	+			1.28 x 10^5	9.10 x 10^2	1.30 x 10^3	< 10	< 10^2
60	wings	bulk	supermarket #1	+		+	2.70 x 10^4	1.30 x 10^2	1.00 x 10^2	< 10	< 10^2

Table S2. Presence of pathogens (*Campylobacter* spp., *Salmonella* spp., and *L. monocytogenes*) and population levels (CFU/g) of hygiene (safety) indicator organisms (APC, *Enterobacteriaceae*, total coliforms, *E. coli*, and staphylococci) in each RTE freshly leafy greens salad sample (n = 40).

s/n	Sample type (salad ingredients)	Retail store	Presence (detection)			Population level (CFU/g)				
			Campylobacter spp.	*Salmonella* spp.	*L. monocytogenes*	APC	*Enterobacteriaceae*	Coliforms	*E. coli*	Staphylococci
1	iceberg lettuce, romaine lettuce, radizio	supermarket #2				5.32 x 10^6	1.32 x 10^3	< 20	< 20	< 10
2	spinach, lollo rosso lettuce, lollo verde lettuce, radizio, rocket, romaine lettuce	supermarket #2			+	2.04 x 10^7	1.03 x 10^4	< 20	< 20	< 10
3	lollo rosso lettuce, lollo verde lettuce, radizio	supermarket #2				6.29 x 10^6	4.90 x 10^2	< 20	< 20	6.00 x 10^2
4	romaine lettuce, escarole, butterhead lettuce	supermarket #3				6.28 x 10^6	9.20 x 10^2	< 20	< 20	4.30 x 10^3
5	iceberg lettuce, romaine lettuce, radizio	supermarket #3				6.19 x 10^6	5.80 x 10^2	< 20	< 20	1.80 x 10^3
6	romaine lettuce, radizio, valerian	supermarket #2				2.38 x 10^6	8.60 x 10^2	< 20	< 20	1.00 x 10^2

7	iceberg lettuce, romaine lettuce, radizio	supermarket #2		1.60 x 10^{5}	2.60 x 10^{2}	< 20	< 20	< 10
8	lollo rosso lettuce, lollo verde lettuce, rocket	supermarket #2		1.42 x 10^{7}	2.20 x 10^{2}	< 20	< 20	< 10
9	rocket	supermarket #2		9.44 x 10^{8}	7.00 x 10^{1}	< 20	< 20	2.00 x 10^{2}
10	spinach	supermarket #2		2.90 x 10^{8}	8.30 x 10^{2}	< 20	< 20	< 10
11	iceberg lettuce, radizio, carrot	supermarket #1		7.00 x 10^{6}	3.60 x 10^{2}	< 20	< 20	< 10
12	wild rocket	supermarket #1		2.02 x 10^{8}	< 10	< 20	< 20	< 10
13	valerian	supermarket #1		1.80 x 10^{8}	< 10	< 20	< 20	< 10
14	iceberg lettuce, lettuce, radizio	supermarket #1		8.10 x 10^{6}	4.30 x 10^{2}	< 20	< 20	< 10
15	lola green, lola red, rocket	supermarket #1		8.40 x 10^{7}	8.80 x 10^{2}	< 20	< 20	1.30 x 10^{3}
16	lollo rosso lettuce, lollo verde lettuce, rocket	supermarket #2		8.00 x 10^{7}	1.20 x 10^{2}	< 20	< 20	< 10
17	spinach, lollo rosso lettuce, lollo verde lettuce, radizio, rocket, romaine lettuce	supermarket #2	+	1.62 x 10^{7}	1.00 x 10^{3}	< 20	< 20	< 10
18	rocket	supermarket #2		4.06 x 10^{8}	1.40 x 10^{2}	< 20	< 20	< 10
19	iceberg lettuce	supermarket #2		1.95 x 10^{7}	2.40 x 10^{2}	< 20	< 20	< 10
20	cabbage, carrot	supermarket #2		1.50 x 10^{6}	2.00 x 10^{1}	< 20	< 20	< 10
21	cabbage, carrot	supermarket #2		2.47 x 10^{6}	< 10	< 20	< 20	< 10
22	romaine lettuce, lettuce salad, curly endive	supermarket #2		1.02 x 10^{7}	1.80 x 10^{2}	< 20	< 20	< 10
23	spinach, lollo rosso lettuce, lollo verde lettuce, radizio, rocket, romaine lettuce	supermarket #2		1.13 x 10^{7}	5.00 x 10^{1}	< 20	< 20	< 10
24	iceberg lettuce	supermarket #2		2.22 x 10^{6}	< 10	< 20	< 20	< 10

25	iceberg lettuce, romaine lettuce, radizio	supermarket #3	+		5.20×10^{7}	2.30×10^{2}	4.00×10^{1}	4.00×10^{1}	< 10
26	spinach, lollo rosso lettuce, lollo verde lettuce, radizio, rocket, romaine lettuce	supermarket #2			1.97×10^{8}	1.80×10^{3}	< 20	< 20	< 10
27	iceberg lettuce	supermarket #2			1.07×10^{6}	< 10	< 20	< 20	< 10
28	romaine lettuce, radizio, valerian	supermarket #2			9.60×10^{6}	8.00×10^{1}	< 20	< 20	1.10×10^{3}
29	lollo rosso lettuce, lollo verde lettuce, rocket	supermarket #2			1.31×10^{8}	1.00×10^{2}	< 20	< 20	< 10
30	cabbage, carrot	supermarket #2			4.10×10^{6}	1.10×10^{2}	< 20	< 20	< 10
31	spinach, lollo rosso lettuce, lollo verde lettuce, radizio, rocket, romaine lettuce	supermarket #2			6.00×10^{7}	3.20×10^{4}	< 20	< 20	1.50×10^{3}
32	iceberg lettuce, romaine lettuce, radizio	supermarket #2			1.00×10^{5}	1.88×10^{3}	< 20	< 20	6.00×10^{2}
33	iceberg lettuce	supermarket #2			1.15×10^{7}	7.00×10^{1}	< 20	< 20	< 10
34	lollo rosso lettuce, lollo verde lettuce, rocket	supermarket #2			1.60×10^{7}	9.60×10^{2}	< 20	< 20	1.00×10^{3}
35	cabbage, carrot	supermarket #2			1.90×10^{7}	2.00×10^{3}	< 20	< 20	< 10
36	iceberg lettuce, white cabbage, radizio, carrot, escarole	supermarket #3			1.12×10^{8}	1.92×10^{4}	< 20	< 20	< 10
37	iceberg lettuce, romaine lettuce, radizio	supermarket #3			6.20×10^{7}	3.10×10^{3}	< 20	< 20	1.00×10^{2}
38	iceberg lettuce, lettuce, radizio	supermarket #3			1.80×10^{7}	1.32×10^{3}	< 20	< 20	2.00×10^{2}
39	lola green, lola red, rocket	supermarket #3			3.22×10^{7}	2.16×10^{3}	< 20	< 20	1.80×10^{3}
40	romaine lettuce, escarole, butterhead lettuce	supermarket #3		+	2.22×10^{8}	1.36×10^{4}	< 20	< 20	1.40×10^{3}

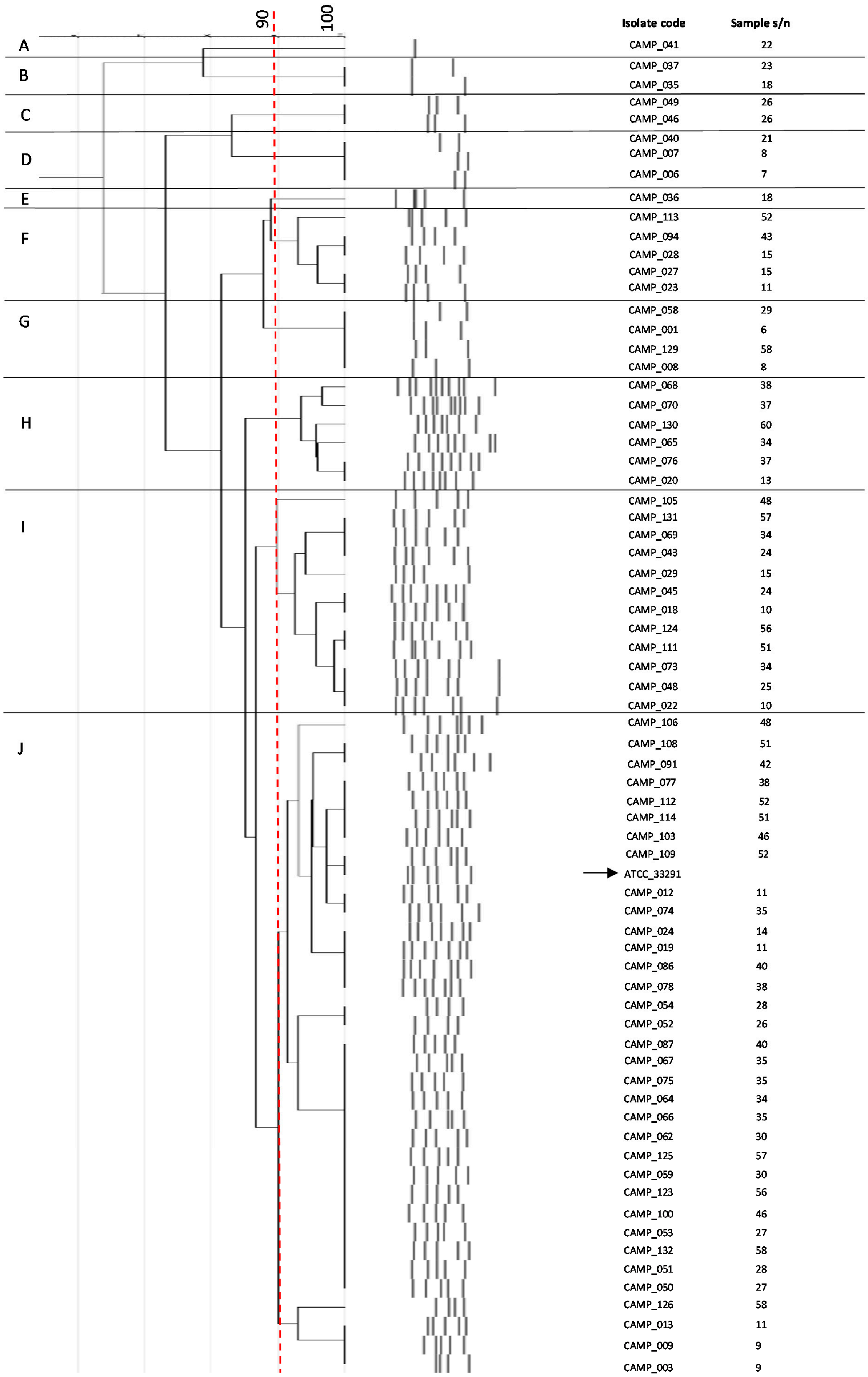

Figure S1. Cluster analysis (dendrogram) based on rep-PCR genotypic patterns of *C. jejuni* raw chicken meat isolates (n = 70). Each separate group (B, C, D, F, G, H, I, and J) includes isolates with coefficient similarity of above 90%. The origin of each isolate (sample s/n) is also indicated. The *C. jejuni* ATCC 33291 strain is indicated with the arrow.

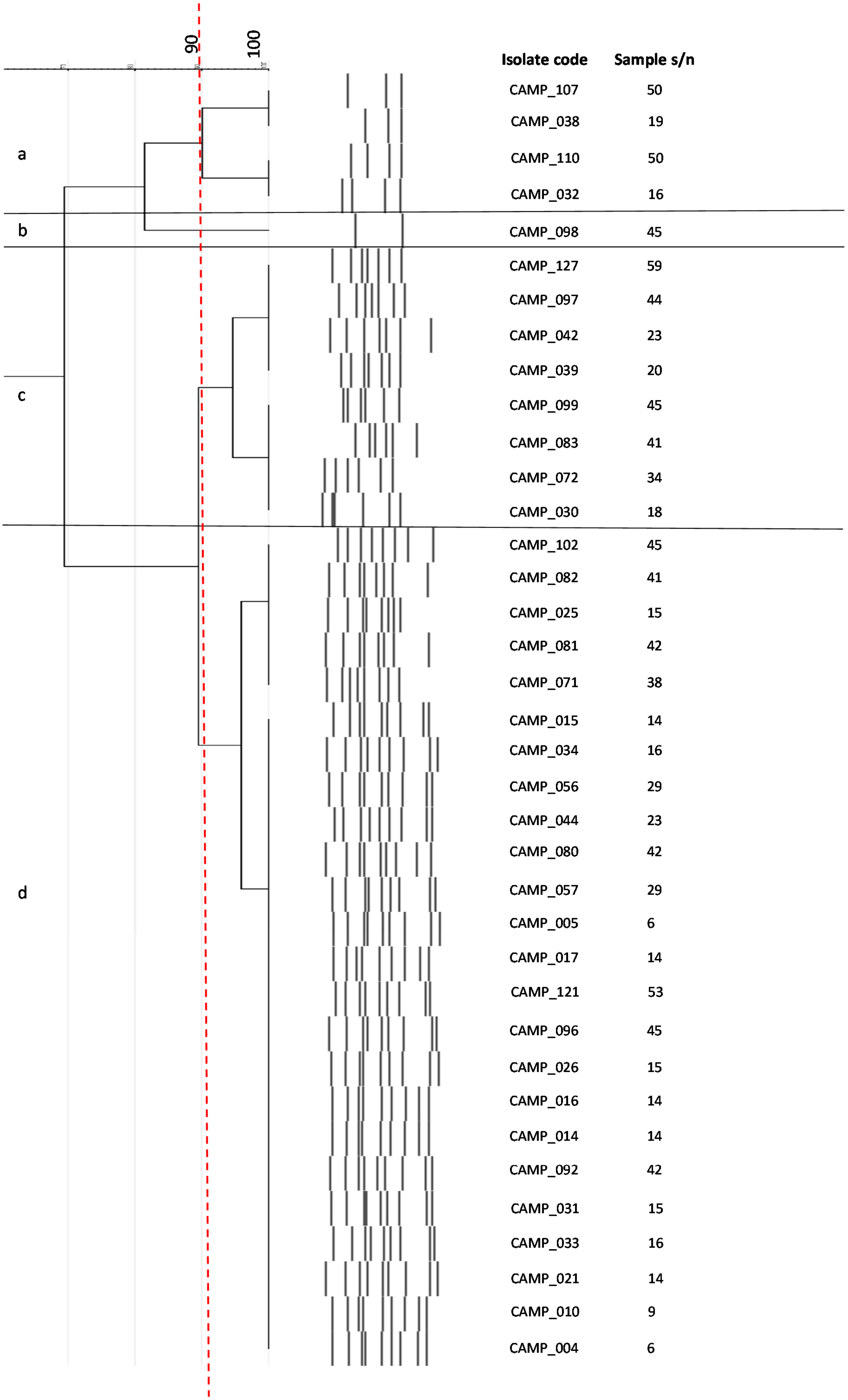

Figure S2. Cluster analysis (dendrogram) based on rep-PCR genotypic patterns of *C. coli* raw chicken meat isolates (*n* = 37). Each separate group (a, c, and d) includes isolates with coefficient similarity of above 90%. The origin of each isolate (sample s/n) is also indicated.

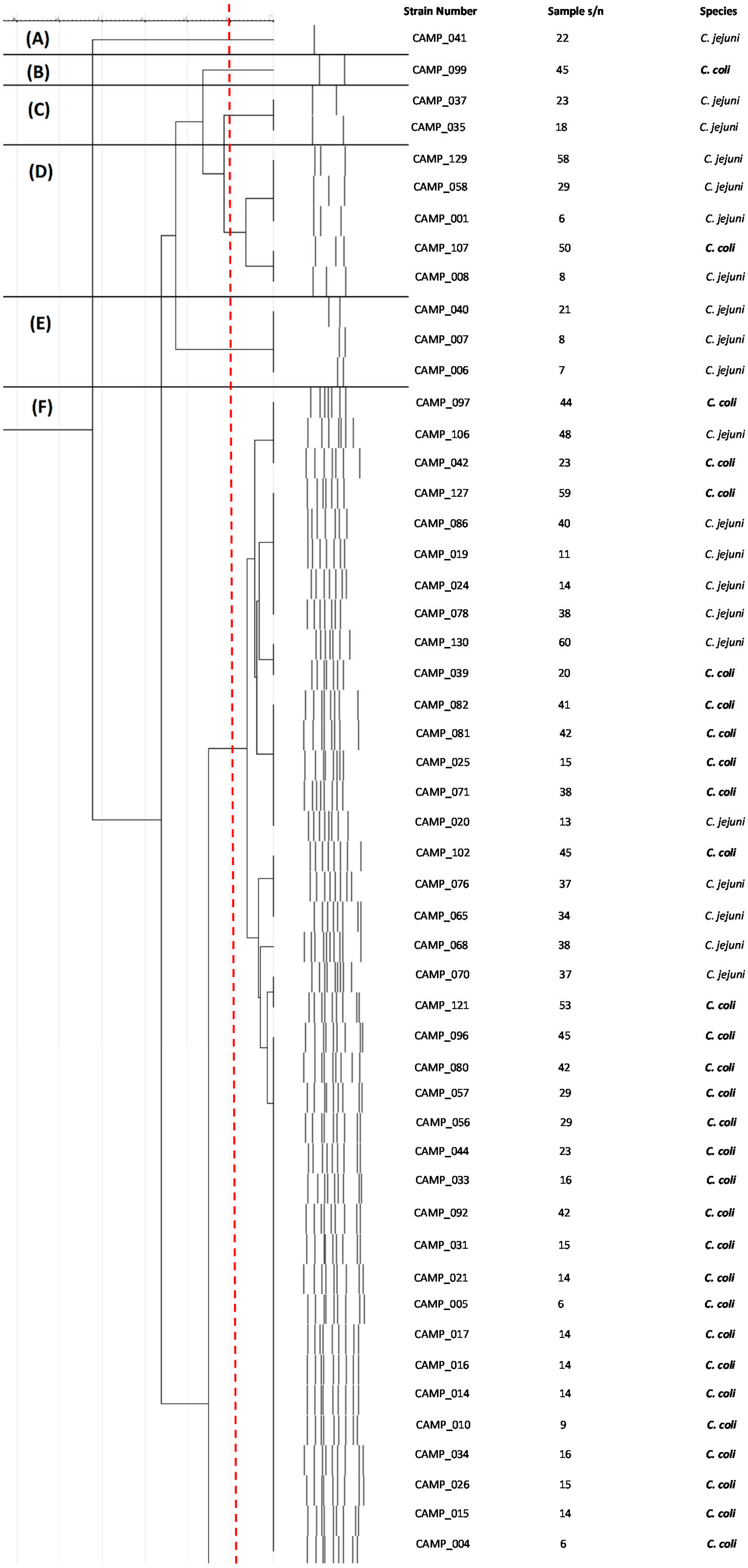

Strain Number	Sample s/n	Species
CAMP_041	22	*C. jejuni*
CAMP_099	45	***C. coli***
CAMP_037	23	*C. jejuni*
CAMP_035	18	*C. jejuni*
CAMP_129	58	*C. jejuni*
CAMP_058	29	*C. jejuni*
CAMP_001	6	*C. jejuni*
CAMP_107	50	***C. coli***
CAMP_008	8	*C. jejuni*
CAMP_040	21	*C. jejuni*
CAMP_007	8	*C. jejuni*
CAMP_006	7	*C. jejuni*
CAMP_097	44	***C. coli***
CAMP_106	48	*C. jejuni*
CAMP_042	23	***C. coli***
CAMP_127	59	***C. coli***
CAMP_086	40	*C. jejuni*
CAMP_019	11	*C. jejuni*
CAMP_024	14	*C. jejuni*
CAMP_078	38	*C. jejuni*
CAMP_130	60	*C. jejuni*
CAMP_039	20	***C. coli***
CAMP_082	41	***C. coli***
CAMP_081	42	***C. coli***
CAMP_025	15	***C. coli***
CAMP_071	38	***C. coli***
CAMP_020	13	*C. jejuni*
CAMP_102	45	***C. coli***
CAMP_076	37	*C. jejuni*
CAMP_065	34	*C. jejuni*
CAMP_068	38	*C. jejuni*
CAMP_070	37	*C. jejuni*
CAMP_121	53	***C. coli***
CAMP_096	45	***C. coli***
CAMP_080	42	***C. coli***
CAMP_057	29	***C. coli***
CAMP_056	29	***C. coli***
CAMP_044	23	***C. coli***
CAMP_033	16	***C. coli***
CAMP_092	42	***C. coli***
CAMP_031	15	***C. coli***
CAMP_021	14	***C. coli***
CAMP_005	6	***C. coli***
CAMP_017	14	***C. coli***
CAMP_016	14	***C. coli***
CAMP_014	14	***C. coli***
CAMP_010	9	***C. coli***
CAMP_034	16	***C. coli***
CAMP_026	15	***C. coli***
CAMP_015	14	***C. coli***
CAMP_004	6	***C. coli***

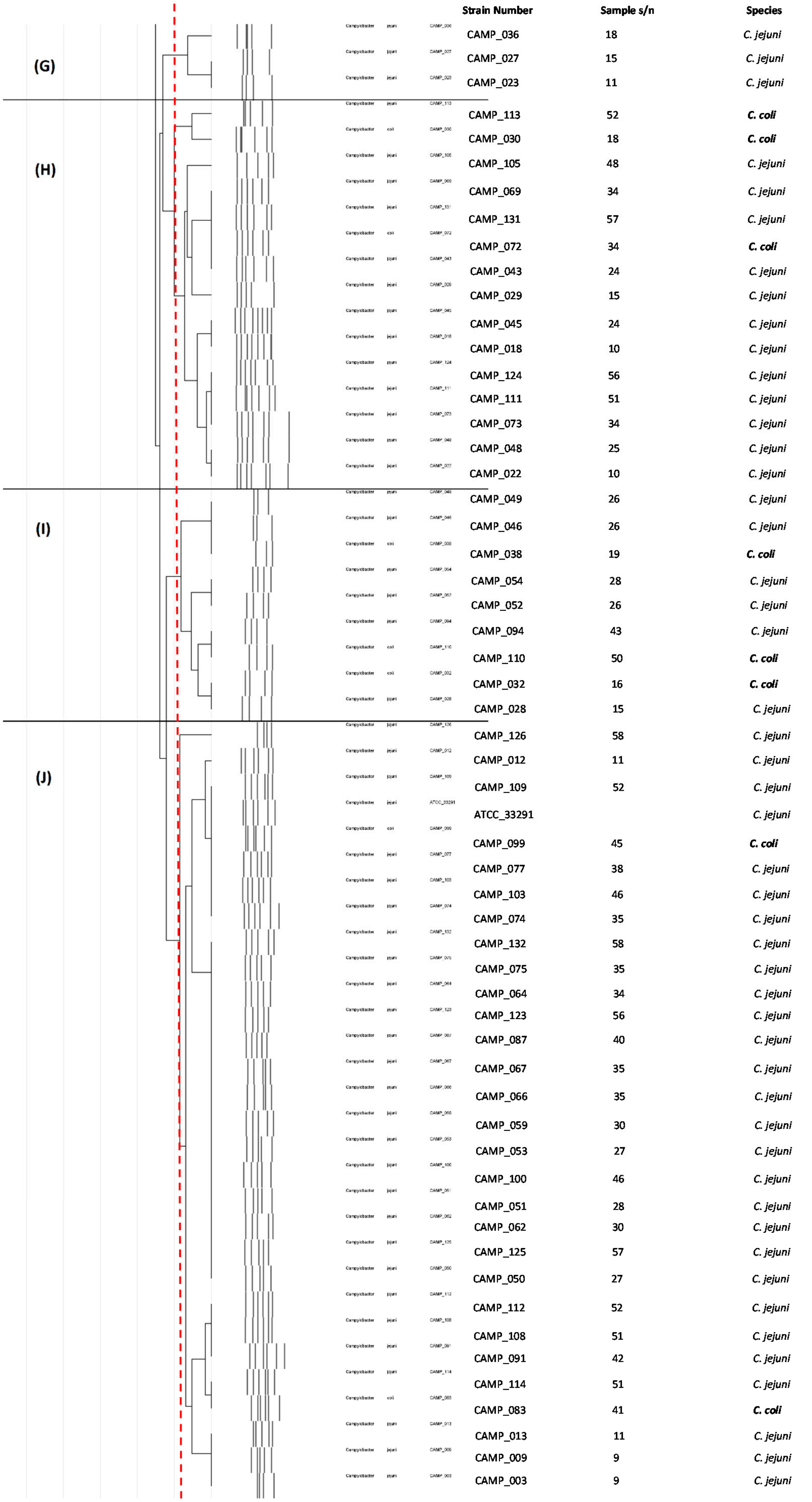

Figure S3. Cluster analysis (dendrogram) based on rep-PCR genotypic patterns of *Campylobacter* spp. raw chicken meat isolates (*n* = 107). Each separate group (B, C, D, E, F, G, H, I, and J) includes isolates with coefficient similarity of above 90%. The origin of each isolate (sample s/n) is also indicated.
